# Laboratory testing of extravascular body fluids: National recommendations on behalf of the Croatian Society of Medical Biochemistry and Laboratory Medicine. Part I – Serous fluids

**DOI:** 10.11613/BM.2020.010502

**Published:** 2019-12-15

**Authors:** Lara Milevoj Kopcinovic, Jelena Culej, Anja Jokic, Marija Bozovic, Irena Kocijan

**Affiliations:** 1 Croatian Society of Medical Biochemistry and Laboratory Medicine, Working group for extravascular body fluid samples; 2 Department of Clinical Chemistry, Sestre milosrdnice University Hospital Center, Zagreb, Croatia; 3 Department of Medical Biochemistry, Haematology and Coagulation with Cytology, University Hospital for Infectious Diseases “Dr. Fran Mihaljević”, Zagreb, Croatia; 4 Medical Biochemistry Laboratory, General hospital Varaždin, Varaždin, Croatia

**Keywords:** harmonization, pleural effusion, pericardial effusion, ascites

## Abstract

Extravascular body fluids (EBF) analysis can provide useful information in the differential diagnosis of conditions that caused their accumulation. Their unique nature and particular requirements accompanying EBF analysis need to be recognized in order to minimize possible negative implications on patient safety. This recommendation was prepared by the members of the Working group for extravascular body fluid samples (WG EBFS). It is designed to address the total testing process and clinical significance of tests used in EBF analysis. The recommendation begins with a chapter addressing validation of methods used in EBF analysis, and continues with specific recommendations for serous fluids analysis. It is organized in sections referring to the preanalytical, analytical and postanalytical phase with specific recommendations presented in boxes. Its main goal is to assist in the attainment of national harmonization of serous fluid analysis and ultimately improve patient safety and healthcare outcomes. This recommendation is intended to all laboratory professionals performing EBF analysis and healthcare professionals involved in EBF collection and processing. Cytological and microbiological evaluations of EBF are beyond the scope of this document.

## Introduction

In medicine, the term “extravascular body fluid” is used with specific reference to all body fluids other than blood. Extravascular body fluids (EBF) analysed in the clinical laboratory comprise cerebrospinal fluid (CSF), serous fluids (pleural, peritoneal and pericardial), synovial fluid, amniotic fluid, drain fluid, semen, urine, dialysate and others. The term “non-standard” body fluids is frequently used in the literature, referring to all body fluids lacking manufacturer’s analytical performance specifications (*i.e.* not listed in the “Intended use” section of the manufacturer’s package insert). However, the term EBF should be distinguished from the term non-standard body fluids, since not all EBF are non-standard samples. For example, urine and CSF, although extravascular by definition, are considered “standard fluids” when analysing total proteins and/or glucose (*1*-*4*).

The composition of EBF is unique and organ/disease dependent. In this context, analysis of EBF can provide useful information in differentiating conditions that caused fluid accumulation and in detecting specific organ involvement (*5*). However, the unique nature and particular requirements accompanying EBF analysis need to be recognized to minimize possible negative implications on patient safety. Extravascular body fluid analysis is compromised by several challenges, including: the appropriate choice of collection containers and storage conditions in case of delayed analysis, analyte stability in EBF, matrix differences possibly affecting the analytical process, lack of quality control (QC) materials *etc.* Furthermore, two additional important issues arise in EBF analysis: analytical performance specifications for “standard fluids” are not transferable to EBF without validation/verification procedures specific for sample type and method used; and lack of reference ranges for EBF require a more prominent involvement of laboratory professionals in results interpretation. Full clinical utility of EBF analysis may be achieved only through harmonization (*2*-*6*).

The objective of this document, prepared by the members of the Working group for extravascular body fluid samples (WG EBFS) of the Croatian Society of Medical Biochemistry and Laboratory Medicine (CSMBLM), is to assist in the attainment of national harmonization of EBF analysis and ultimately improve patient safety and healthcare outcomes. The role of laboratory professionals is not only to ensure reliable EBF test results, but also to encourage the appropriate use of EBF analyses and provide guidance in results interpretation. Thus, this recommendation is intended primarily to all laboratory professionals performing EBF analysis, but also to all healthcare professionals involved in EBF collection and processing.

## Background and scope

Recognizing the importance of harmonization in the field of laboratory medicine, the CSMBLM has so far published several national recommendations (*7*-*11*). This recommendation is the first of a series of recommendations that will address preanalytical, analytical and postanalytical issues in analysis of various EBF.

This document is based on results of the Croatian survey on laboratory EBF testing which gave an insight on the current state of procedures used in EBF analysis in Croatia. The main findings of the survey showed that procedures used in EBF laboratory testing across Croatia are not harmonized and deviations from desirable procedures were detected in all phases (preanalytical, analytical and postanalytical) of EBF analysis (*12*). Furthermore, two guidelines issued by the Clinical and Laboratory Standards Institute (CLSI) (*i.e.* “Analysis of body fluids in clinical chemistry. Approved guideline. CLSI document C49-A”, and “Body fluid analysis for cellular composition: Approved guideline. CLSI document H56-A”) were used as a framework while writing this document (*5*, *13*). Finally, a thorough review and critical assessment of relevant evidence available from scientific literature was conducted. PubMed, Scopus and Google Scholar were searched using the following key words/terms: body fluid analysis, pleural effusion, pericardial effusion, peritoneal effusion, ascites, peritoneal fluid analysis, pericardial fluid analysis, ascites analysis. The search was limited to articles in English language pertaining to human subjects. Relevant articles were identified by title and abstract screening, full texts were retrieved and relevance of the content was critically assessed. After initial search, a more specific literature search was conducted using specific terms (*e.g.* Light’s criteria, pleural fluid cholesterol, albumin gradient in ascites *etc.*). Finally, relevant articles referenced in articles retrieved in the initial search were used.

The recommendation begins with a chapter addressing validation of methods used in EBF analysis, and continues with specific recommendations for serous fluids analysis (comprising pleural, peritoneal and pericardial fluid), since these fluid types are analysed in almost all laboratories in Croatia (*12*). It is organized in sections referring to preanalytical, analytical and postanalytical aspects of serous fluid analysis. Specific recommendations are presented in boxed, followed by explanations and interpretative data derived from relevant guidelines and literature. Based on the strength and availability of scientific evidence retrieved, recommendations in boxes were categorized as Class 1 (moderate recommendation) and Class 2 (limited recommendation).

Serous fluids, like all samples processed in the laboratory, should be handled according to institutional and/or national health and safety regulations in order to minimize potential health risks (*5*). Although EBF analysis includes other speciality areas besides chemistry and haematology, cytological and microbiological evaluations are not performed in Croatian medical biochemistry laboratories, and thus are beyond the scope of this document.

## 1. Validation of analytical performance specifications for EBF

Laboratories should inspect the manufacturer’s performance specifications for methods used in EBF analysis. If performance specifications for EBF sample types analysed are included, a verification procedure should be performed (*2*). If method performance specifications are not provided by the manufacturer, and an assay is to be extended for application to EBF analysis, method validation should be performed for each combination of EBF type and assay used (*2*-*5*, *14*-*16*). Since guidance for analytical verification/validation of EBF is limited, each laboratory should perform EBF verification/validation procedures relying on available procedures for “standard fluids” (e.g. available CLSI documents). All verification/validation procedures should be documented (Class 1).

Extravascular body fluids analysis is usually performed using assays intended for “standard samples” (*i.e.* serum, plasma, whole blood, urine) on automated analysers, which makes it widely available and relatively inexpensive (*2*-*6*, *14*). Since methods manufacturers in general do not provide performance specifications for EBF, the use of methods for “standard” fluids in EBF analysis is considered a method modification and requires validation according to the specific laboratory’s accreditation/professional regulations (*2*, *15*, *16*). This issue was recognized by the CLSI and also by the College of American Pathologists (CAP) accreditation requirements (*5*, *16*, *17*).

The first step in the verification/validation procedure is selection of tests to be verified/validated. The most prevalent EBF types and analyses, with established clinical utility, selected in collaboration with clinicians, should be verified/validated. A retrospective analysis of data on serous fluid types and analyses requested, from the laboratory information system (LIS) if available, should provide an insight of the most frequently analysed EBF types and analyses in order to plan appropriate verification/validation procedures. Assays that do not add value to patient management (*i.e.* not mentioned in this document) should be discouraged and discontinued (*2*-*6*, *18*, *19*).

The approach to EBF verification should include the following: precision, trueness, analytic measurement range (AMR)/reportable range evaluation, and, if applicable, verification of manufacturer supplied reference intervals. Procedures and principles for method verification of “standard fluids” should be applied to EBF verification (*e.g.* available CLSI documents) (*2*-*6*, *18*, *20*).

The analytical validation of methods used in EBF analysis is more complex and labour intensive mainly due to the lack of analytical performance claims provided by the manufacturer and lack of commercially available matrix-matched QC materials for all EBF (*2*, *4*, *20*). Performance acceptance criteria should be adopted from the standard fluid’s verification procedure (*e.g.* when validating the albumin method in a peritoneal fluid sample, the performance claims provided by the manufacturer for serum albumin should be adopted) (*2*, *5*). If those criteria are not met, the laboratory should evaluate the impact of the obtained deviations from acceptable criteria on clinical decision limits and results’ interpretation. Accordingly, the utilization of such “standard” assays for EBF analysis should be authorized or rejected (*2*, *5*). For validation purposes, the laboratory should collect EBF sample leftovers obtained after routine analysis. Depending on frequency of EBF types analysed, this process might last a prolonged period of time and the storage of such samples (refrigerated or frozen) might be necessary. Since the stability of analytes in EBF samples is largely unknown, stability studies should be undertaken before validation (see 3.1.3 Postanalytical phase) (*2*, *5*, *18*-*20*).

The protocol for EBF validation should include evaluation of precision, trueness, analytical sensitivity, analytical specificity (interferences), AMR/reportable range, and, if necessary carry-over, clinical sensitivity and clinical specificity. All validation experiments should be performed similarly to “standard fluid” protocols (*2*, *5*, *18*-*20*).

The most important issue contributing to potential unreliability of EBF analysis using assays intended for “standard” sample types is the EBF matrix effect. The matrix effect refers to differences in composition between EBF and serum/plasma. Variations in pH, electrolytes, protein and lipid concentrations found in specific EBF sample types can be marked, influencing the change in physical and chemical properties of the EBF and affecting the preanalytical and analytical phase of EBF analysis (*2*, *5*, *20*, *21*). The EBF matrix effect is investigated concomitantly to trueness and/or AMR evaluation (*i.e.* in mixing studies). Different approaches might be applied depending on the availability of EBF samples with high/low analyte concentration. For example, if one EBF sample with low and one with high analyte concentration are available, their mixtures with increasing concentrations of the target analyte across the AMR should be produced and a 5-point curve should be generated. Alternatively, a standard sample type, standard solution or calibrator with known high concentration of the target analyte are used for spiking an EBF sample with low analyte concentration. Matrix interference is evaluated by comparing measured results to expected target analyte concentrations. In either case, if the reproducibility is comparable to standard samples and/or dilution ratios are recovered, the existence of matrix effect can be reasonably excluded (*2*, *5*, *17*). If no EBF matrix effect on a specific assay is found, manufacturer’s specifications for validated sample types for assay specificity and interfering substances, might be employed (*17*, *19*). If interference due to matrix effect cannot be excluded, the EBF test result should be reported only if accompanied with a comment clearly stating the analytical limitations of the method used emphasizing the need for interpreting results in the clinical context (*2*, *20*).

Automated analysers used for EBF total and differential cell counting with a built-in body fluid (BF) mode offer an automated solution for cell counting and differentials in various body fluids (see Appendix 1). These systems are accompanied with manufacturer’s specifications for each validated EBF type. Guidelines to help laboratories in performing verification of EBF cell counting using automated haematology analysers are available and will not be discussed here in further details (*15*, *22*). If an EBF type not listed by the manufacturer is to be analysed by the laboratory, a validation should be performed, including the evaluation of precision, trueness, analytical sensitivity, analytical specificity (interferences), AMR/reportable range and carry-over (*13*, *15*, *22*).

## 2. Quality control and proficiency testing for EBF assays

Quality control testing for EBF analyses should be instituted in accordance with quality management strategies already present in the laboratory. The QC materials (independent if commercial or not) should be analysed in the same manner as routine EBF samples (*6*, *16*). Procedures to be followed in case of unacceptable QC results should be instituted and documented.

If commercial proficiency testing (PT) programmes are available for EBF analyses, laboratories should participate (*5*). The minimal recommended frequency is one cycle yearly. When PT programmes are not available for specific EBF analytes, inter-laboratory comparison programmes should be instituted (Class 1) (*6*).

If matrix-matched QC materials are not commercially available, EBF samples collected in the laboratory might be used. Samples designated for QC analysis should possibly cover the AMR and decision limits. The extended stability of analytes in EBF samples under different storage conditions should be verified by individual laboratories prior to implementation (*5*, *19*).

If cell counts and differentials in EBF samples are performed using automated methods, appropriate QC should include the performance of a background count (check) of the analyser fluidic system. The limits of such background count should be defined by individual laboratories depending on the EBF type tested (*13*, *15*).

Proficiency testing programmes from independent providers are available for urine analyses and certain CSF, semen and synovial fluid tests. However, the availability of PT materials for all EBF types is limited. If PT is not commercially available, individual laboratories are strongly encouraged to organize inter-laboratory comparisons. Both PT and inter-laboratory comparisons should encompass the organisation and evaluation of at least one cycle yearly, with the participation of two or more laboratories. Participating laboratories in inter-laboratory comparisons are responsible for defining acceptable performance criteria for all the analytes tested (*13*, *15*).

Quality control, PT and inter-laboratory comparison results should be reviewed and acted upon in a timely manner in case of unsatisfactory performance, as per laboratory procedures, to ensure reliability of results reported. All QC procedures should be documented according to the QC assurance policy instituted in the laboratory (*16*).

## 3. Serous fluids analysis

### 3.1 Pleural fluid

Physiologically, only a small volume of pleural fluid (< 10 mL) is formed by plasma ultrafiltration in the capillary endothelium of the pleural cavity. Its function is to lubricate the motion of pleural membranes against each other. In case of pleural fluid accumulation, due to imbalance in fluid formation and/or absorption, a pleural effusion is formed (*5*, *6*, *14*, *23*, *24*). Pleural effusions are most frequently caused by congestive heart failure, liver cirrhosis, pulmonary infections, malignancy or pulmonary embolism; and are usually diagnosed by physical examination and chest radiography. In case of pleural effusion without definitive diagnosis, the collection of pleural fluid for laboratory analysis is indicated (*5*, *6*). It is estimated that based on patient history, physical examination and pleural fluid analysis the clinician could diagnose the specific disease underlying pleural fluid accumulation in 95% of cases (*24*).

#### 3.1.1 Preanalytical phase

##### 3.1.1.1 Test request form and test ordering

The test request form for pleural fluid analysis should adhere to accreditation requirements (*16*). It should contain the patient’s name, surname, sex, date of birth and unique identifier (*e.g.* health insurance number), collection date and time, the working diagnosis, hospital ward, identification of the ordering physician and its contact details, identification of the clinical staff that performed collection. Tests requested and any clinically relevant information (*e.g.* diuretic therapy) to facilitate results interpretation should be clearly indicated on the test request. Additionally, the collection procedure (*i.e*. thoracentesis or pleural tap), collection site and anatomic origin of the sample should be clearly stated (Class 1) (*7*, *25*).

An appropriate test request form should accompany pleural fluid samples sent to the laboratory. Optimizing the test request form to include only clinically useful tests with available interpretive information will improve pleural fluid analysis and interpretation (*4*).

Unusual requests are often ordered either accidentally or inappropriately. In such cases the laboratory should contact the requesting clinician to establish if the order is justified and clinically meaningful (*i.e.* why the test was ordered, how results will be interpreted) or it should be cancelled (*2*, *4*).

##### 3.1.1.2 Patient and sample identification

Pleural fluid samples should be labelled in the presence of the patient (preferably immediately prior to collection), with at least two unique identifiers (*i.e.* name, date of birth or health insurance number), location, date and time of collection and anatomic site of collection (Class 1) (*5*, *7*).

Sample containers not labelled properly (or unlabelled) should not be accepted for analysis (*5*). Sample rejection should be documented in the laboratory, stated on the patient’s report and communicated to the ordering physician.

##### 3.1.1.3 Pleural fluid samples collection and handling

The collection container and sample handling procedures *(i.e.* transport and processing) used for pleural fluid analysis should be dictated by the tests ordered (*5*, *17*, *26*, *27*). Ethylenediaminetetraacetic acid (EDTA) anticoagulated tubes should be used for total and differential cell counts, while pleural fluid samples for biochemical analyses should be collected in heparin anticoagulated tubes. Alternatively, if transport conditions are met, pleural fluid samples for cell counts and biochemical analyses might be collected in plain tubes, *i.e.* tubes containing no additives (*13*, *22*, *26*, *28*, *29*).

The pleural fluid sample should be transported to the laboratory at room temperature immediately after collection (within one hour for cell count and differentials). It should be processed promptly upon receipt.

For interpretative purposes, a serum sample should be collected within one hour from pleural fluid collection and sent to the laboratory (Class 1) (*6*, *13*, *18*, *30*).

Pleural fluid samples are usually collected on clinical wards or operating rooms, by experienced clinical personnel, using a procedure called thoracentesis, often as image-guided thoracentesis (*e.g.* ultrasound-guided). Thoracentesis is performed for either diagnostic or therapeutic purposes (or both). It involves pleural fluid aspiration from the pleural space using a needle. Although not under direct laboratory supervision, the collection technique used might greatly affect EBF analysis results. Thus, clinical practice guidelines should be followed in order to standardize procedures used in thoracentesis (*13*, *18*, *22*, *27*, *31*).

Large syringes (with or without needle) used for thoracentesis, containing pleural fluid sample, are not acceptable containers. Large sample volumes (usually more than 20 mL) collected in thoracentesis syringes should be transferred into appropriate containers at the collection site before transportation to the laboratory. This is particularly important in avoiding sample clotting (particularly if the sample is haemolysed) (*13*, *22*, *26*, *28*, *29*).

Tubes containing anticoagulants should be mixed according to manufacturer’s instructions to ensure proper mixing and avoid clot formation (*28*). Tubes for biochemical analyses should be centrifuged prior to analysis as *per* serum centrifugation conditions (*14*).

For pH measurement, pleural fluid samples should be collected anaerobically, in syringes containing lyophilized, balanced lithium heparin (as for blood gas analysis) and transported to the laboratory immediately. Pleural fluid samples for pH determinations should be collected and analysed similar to whole blood samples for blood gas analysis. Contemporary blood gas analysers are able to measure not just blood gas parameters but also related measurements (*i.e.* electrolytes and metabolites). However, due to limited preanalytical data, the feasibility of performing all pleural fluid analyses from pleural (as well as other serous) fluid samples collected in lyophilized syringes is unclear (*5*, *6*, *9*, *22*).

Acceptable pleural fluid sample volumes should be submitted for analysis. Minimum sample volumes should be defined according to the organization of each individual laboratory (*e.g.* minimum 3 mL for total and differential cell count if EDTA containers are used; minimum 2 mL for total and differential cell counts if containers without additives are used; minimum 3 mL for biochemical analysis if heparin containers are used; minimum 2 mL for biochemical analysis if containers without additives are used; minimum 1 mL for pH determination). If limited (or insufficient) sample volumes (less than those acceptable) are submitted for analysis, test priority should be established in consultation with the ordering clinician (*6*, *27*).

##### 3.1.1.4 Assessing sample quality

The quality of the pleural fluid sample should be inspected before analysis to avoid instrument failures and/or measurement errors. The laboratory should recognize and document the possible impact of haemolysis, lipemia, icterus and extreme pH values present in the sample on measurement results. Grossly haemolysed and clotted samples may affect results’ accuracy and should not be considered suitable for analysis. Exceptions to the rule of rejecting unsuitable samples should be defined and documented (Class 1) (*2*, *5*, *13*, *14*).

Pleural fluid samples should be visually checked before analysis. Such samples might present with altered fluid tension, viscosity and/or miscibility due to the fluid’s matrix effect. These alterations might cause inaccurate sample aspiration/dispensing into the reaction mixture, inappropriate mixing or incomplete cleansing of the dispensing and/or reaction mechanisms, frequently not recognized by the instrument used. In case of viscous samples, pre-treatment procedures (*e.g.* re-centrifugation, dilution) should be considered (*5*, *20*).

Although not sufficiently accurate to measure pleural fluid pH, a pH meter or pH litmus paper (pH indicator stick) might be used to broadly indicate pleural fluid pH values in case of suspected extreme pH. The application of urine dipsticks in assessing pleural fluid extreme pH values poorly investigated in the literature (*32*, *33*).

#### 3.1.2 Analytical phase

##### 3.1.2.1 Pleural fluid appearance

Pleural fluid appearance should be determined upon sample acceptance and before centrifugation. Pleural fluid appearance should not be used as the sole criterion for differentiation of exudates (caused by localized disorders) from transudates (caused by systemic disease) (Class 1) (*23*, *27*, *34*).

**Figure 1:**
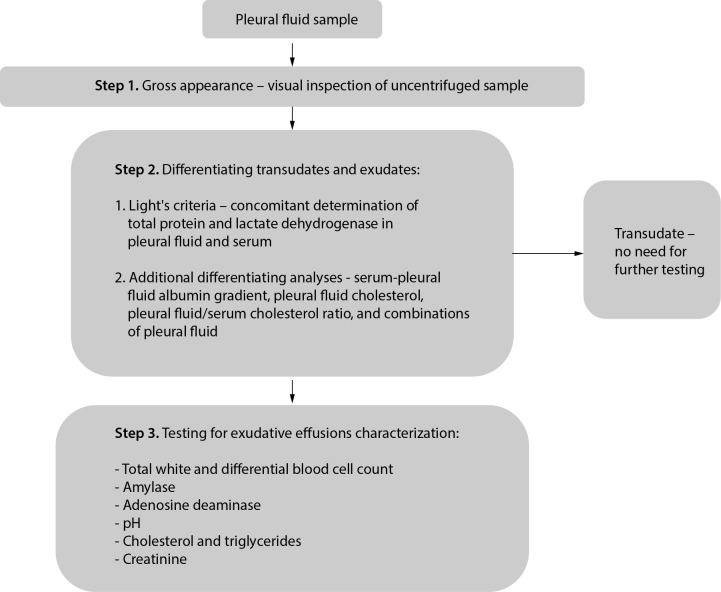
Algorithm for pleural fluid testing

The initial step in laboratory investigation of pleural fluid is determination of fluid’s appearance. Although not definitive, it is straightforward and might suggest the effusion’s aetiology (Table 1 and Figure 1). Appearance of pleural (as well as other serous) fluid samples may be estimated equally well from plain and/or anticoagulated tubes, prior to centrifugation.

**Table 1:** Possible interpretation of pleural fluid appearance

**Appearance**	**Possible clinical significance**	**References**
TRANSUDATES		
Clear, light yellow, odourless, non-viscous	No need for further laboratory testing	22,26,34
EXUDATES		
Cloudy, turbid, purulent, pronounced clotting tendency	Infection, empyema (due to anaerobic bacteria if putrid odour is present)	22,26,34
Blood tinged or bloody	Trauma, malignancy, pulmonary infarction, aortic aneurysm rupture, tuberculosis, pancreatitis	
Green white, turbid	Rheumatoid pleuritis	
Turbid, milky and/or bloody	Chylous effusion (leakage from the thoracic duct, trauma or idiopathic)	
Milky or green, metallic sheen	Pseudochylous effusion (chronic effusions in rheumatic pleuritis or tuberculosis) or bilio-pleural fistula	
Anchovy brown, chocolate	Rupture of amoebic liver abscess, long standing bloody effusion	
Black	Spores of *Aspergillus niger*	

A traumatic tap might be differentiated from other causes of bloody pleural fluid. It is characterized by uneven blood distribution or formation of small blood clots. A haematocrit > 0.500 L/L suggests a true haemothorax, usually present in chest trauma (*6*, *22*, *23*). Turbid, milky and/or bloody pleural fluid samples should be centrifuged. If the remaining supernatant is clear, this suggests the presence of increased cellular elements or debris *(i.e.* presence of empyema). If turbidity remains after centrifugation, it is most likely that a chylous or pseudochylous effusion is present and lipid analysis is warranted (*6*, *22*-*24*, *34*).

##### 3.1.2.2 Differentiating transudates and exudates

Light’s criteria should be applied to differentiate transudative from exudative pleural effusions. They include the concomitant measurement of total protein and lactate dehydrogenase (LD) in pleural fluid and serum. Accordingly, an exudative effusion meets at least one the following criteria:

- pleural fluid/serum protein ratio > 0.5, and/or
- pleural fluid/serum LD ratio > 0.6; and/or
- absolute pleural fluid LD activity > 2/3 of the serum upper reference limit (URL) (Class 1) (*34*-*36*).

In situations when Light’s criteria are not definite (*e.g.* in patients receiving diuretic therapy; or when clinical symptoms suggest a transudative, but Light’s criteria are indicative of exudative effusion), serum-pleural fluid albumin gradient, pleural fluid cholesterol, pleural fluid/serum cholesterol ratio, or combinations of pleural fluid cholesterol, pleural fluid LD and pleural fluid protein may help in differentiation (Class 1) (*37*). Pleural fluid and serum measurements used for calculation of ratios and gradients should be performed using the same method (Class 2).

Laboratory evaluation of pleural fluid samples is primarily directed towards differentiation of transudative and exudative effusions. This approach greatly simplifies the diagnostic process allowing early exclusion of unnecessary investigations and aiding identification of the underlying mechanism of pleural fluid formation. Transudative pleural effusions are caused by non-inflammatory systemic processes and usually require no further diagnostic procedure. Exudative effusions are indicative of an inflammatory or malignant process of the pleura, and require more extensive laboratory testing to identify the cause of fluid accumulation (*14*, *23*, *34*).

Total protein measurement in pleural fluid samples, as the sole criterion for differentiation, should be abandoned due to high misclassification rates (*2*, *23*). Instead, Light’s criteria are considered the most reliable method to differentiate transudates from exudates. Total proteins and LD are readily measured on automated analysers (using spectrophotometric methods), the criteria are easy to remember and reliable in exudates identification (with a sensitivity and specificity of 98% and 74%, respectively) (Figure 1) (*23*, *36*, *38*). However, Light’s criteria misclassify about 25% of transudates as exudates, particularly in patients receiving diuretic therapy or in cases of higher erythrocyte count (> 10 x10^9^/L) present in the sample (*34*, *36*, *39*).

The albumin gradient has been shown to yield similar diagnostic performances to Light’s criteria in the differentiation of transudates and exudates (with a sensitivity and specificity of 63% and 94%, respectively) (*23*, *36*, *39*-*41*). It should be emphasized that albumin gradient should not be used as the sole criterion for discriminating transudates and exudates because of its high misclassification rates (37%) (*39*).

Pleural fluid cholesterol is useful in differentiating transudates from exudates. The cut-off suggested in Appendix 2 identifies pleural exudates with 89% sensitivity and 81% specificity (*37*). A pleural fluid/serum cholesterol ratio > 0.3 can differentiate exudates with 92% sensitivity and 81% specificity, displaying similar diagnostic performances to Light’s criteria (*5*, *18*, *37*).

Pleural fluid and serum measurements used for calculation of ratios and gradients should be obtained using the same method. Additionally, according to available literature data, the determination of analytes in pleural fluid (*e.g.* total protein and albumin) is not analytically challenging as is the case with ascites (see 3.3.2.2 Differentiating peritoneal effusions).

Other combinations of paired and triplet tests, performed only in the pleural fluid sample, demonstrated similar diagnostic accuracies compared to Light’s criteria. The advantage of this approach is its cost-effectiveness without affecting diagnostic accuracy (*i.e.* no concomitant blood sample is required) (*22*, *37*, *42*).

N-terminal pro-brain natriuretic peptide (NT-proBNP) determination in pleural effusions should be confined to cases with clinically suspected cardiac pleural effusions that meet exudative Light’s criteria, specifically when effusions are bloody or following diuretic therapy. Heart failure (HF) accounts for 80% of transudative pleural effusions. N-terminal pro-brain natriuretic peptide concentrations are increased in patients with HF. A NT-proBNP concentration > 1500 ng/L in pleural fluid achieves 94% sensitivity and 89% specificity for the diagnosis of HF (*43*). The pooled diagnostic performances of NT-proBNP from 14 studies investigating pleural effusions caused by HF were 92% sensitivity and 88% specificity (*44*). Since a significant correlation between serum and pleural fluid NT-proBNP concentrations has been confirmed and both measurements show equivalent diagnostic performances, the measuring NT-proBNP in pleural fluid samples does not add to the diagnostic process of HF (*18*, *39*, *45*, *46*).

##### 3.1.2.3 Analysis of exudative effusions

Laboratories are responsible for encouraging appropriate testing in pleural fluid analysis. Only tests with documented clinical usefulness for the evaluation of exudative effusions are recommen-ded. Test results obtained from exudative effusion evaluation should always be correlated with clinical symptoms and suspected diagnosis (Figure 1).

###### Total white and differential cell count

Criteria for the selection of the appropriate cell counting method should be: frequency of test requests received for pleural fluid cell counts, availability of technical equipment and competence/experience of laboratory personnel (*13*, *48*).

Total white blood cell (WBC) count should not be determined for the differentiation of pleural transudates and exudates (*34*, *47*, *48*). However, total WBC and differential cell count should be determined in exudative pleural effusions as an aid in characterizing inflammatory disorders (*38*, *48*). Total WBC and differential cell count in pleural fluid samples should be performed using automated cell counting methods, or alternatively manual microscopy (*i.e.* using a haemocytometer) (Class 1).

Although total WBC count has limited value and is not recommended for differentiating transudates from exudates, it was shown that exudative effusions have a WBC count of > 1000 x10^6^/L, while those transudates present with WBC counts of < 1000 x10^6^/L (*47*, *48*). Cell types that might be found in pleural fluid include neutrophils, lymphocytes, plasma cells, monocytes and macrophages, mesothelial cells or malignant cells (*6*, *34*). Differential cell count has limited value but might help in narrowing the diagnosis of disorders causing exudative effusions (Table 2 and Appendix 2) (*27*, *45*, *48*). Pleural fluid eosinophilia (> 10% of total WBC) develops within hours in cases of spontaneous pneumothorax, while in cases of traumatic or haemorrhagic pleural effusions it develops after 10 to 14 days. Eosinophilia in pleural effusions following pleural trauma and haemothorax persists until effusion resolution and correlates well with peripheral blood eosinophilia (*24*, *49*). The most common causes of neutrophilia, lymphocytosis and eosinophilia are presented in Table 2. Since neutrophilia and lymphocytosis are found in > 10% and > 30% of transudates, respectively, results should be interpreted in conjunction with clinical symptoms (Table 2).

**Table 2:** Disorders related to neutrophilia, lymphocytosis and eosinophilia in pleural fluid

**Neutrophilia**	**Lymphocytosis**	**Eosinophilia**	**References**
Bacterial pneumonia (parapneumonic effusion)	Tuberculosis	Pneumothorax	34,38,47,48
Pulmonary infarction	Viral infection	Malignancy	
Pancreatitis	Malignancy	Trauma (haemothorax)	
Early tuberculosis	Chylothorax	Pulmonary infarction	
Usually found in > 10% transudates	Rheumatoid pleuritis	Congestive heart failure	
	Usually found in > 30% transudates	Parasitic, fungal infection	

Monocytes/macrophages are the predominant cell population found in pleural fluids (60-80%). Conversely, basophils are rarely found in pleural effusions. The clinical significance of both these cell types is largely unknown (*13*). Mesothelial cells are normally found in pleural effusions and comprise up to 5% of nucleated cells. They might be increased in cases of pneumonia, pulmonary infarction, and malignant disorders. Their clinical significance is in excluding tuberculosis; thus if > 5% mesothelial cells are present in the pleural fluid sample, tuberculosis is not likely (*13*).

Atypical cells (tumour cells, atypical and reactive mesothelial cells *etc.*) found during morphological assessment of pleural fluid sample (either by haemocytometer or evidenced by significant difference in total nucleated cells (*i.e.* all cells containing nucleus) and leukocytes by automated counting) should be addressed by informing the ordering physician in order to suggest the need for cytological evaluation (see 3.1.3 Postanalytical phase).

###### Amylase

Amylase (AMY) should be determined in pleural effusions if pancreatitis, malignancy, oesophageal rupture, pancreatic pseudocyst and liver cirrhosis are suspected (Class 1) (*5*, *22*, *50*-*52*).

High AMY activity in pleural effusions refers to AMY activity that exceeds the serum reference interval or fluid-to-serum amylase ratio > 1. Although not recommended, lipase activity might be also measured as an aid in the differentiation of high AMY activities. In case of oesophageal rupture or malignancy, AMY activities will be high due to higher salivary AMY isoenzymes (with low lipase activities). Pancreatic AMY isoenzymes will be higher in pleural effusions related to pancreatic diseases (with high lipase activities). The interpretation of high AMY activities in pleural effusions is presented in Appendix 2 (*5*, *18*, *24*, *52*).

###### Adenosine deaminase

Pleural fluid adenosine deaminase (ADA) is an accurate and useful indicator of tuberculous pleurisy. It should be measured in patients with suspected tuberculous effusions to differentiate tuberculous and malignant effusions (Class 2) (*53*-*55*).

Diagnosis of tuberculous pleurisy is established combining pleural fluid microscopic examination and pleural fluid cultures. However, positive pleural fluid cultures are found only in 36% of patients with tuberculous effusions, which combined with non-specific symptoms renders it difficult to differentiate tuberculous pleurisy from malignant pleural effusions (*23*, *53*, *54*). Pleural tissue biopsy is considered the most reliable confirmation method for tuberculous pleural exudates. However, pleural fluid biomarkers have been investigated as an alternative to this invasive diagnostic procedure. Adenosine deaminase is released during immune response to *Mycobacterium tuberculosis* in the pleura and is easily measured by automated methods in pleural fluid samples. Its activities are significantly higher in tuberculous pleuritis. Recent meta-analyses showed that an ADA cut-off of ≥ 40 U/L yields an overall sensitivity and specificity of 92% and 90%, respectively. However, ADA predictive values depend on local tuberculosis prevalence and falsely higher ADA activities can be found in parapneumonic effusions and empyema. Thus, ADA results should be interpreted in conjunction with clinical findings, microbiologic examination and pleural biopsy results (*53*-*55*).

###### pH

Pleural fluid pH should be determined in patients with parapneumonic effusions. A pH > 7.30 indicates the need for a pharmacological approach, while a pH < 7.20 (and/or pleural fluid LD > 3 times the serum URL) strongly suggests a complicated parapneumonic effusion requiring surgical evacuation (Class 2) (*5*, *18*, *22*, *23*).

Pleural fluid pH should be determined using a blood gas analyser. Indicator papers and pH meters are inaccurate for clinical decision making (*14*, *24*, *32*, *33*). Other blood gas parameters measured by analysers should not be reported (see 3.1.1.3 Pleural fluid samples collection and handling*)*. If the measured pleural fluid pH is low (< 7.35), arterial pH might be determined to rule out systemic acidosis (*5*, *23*).

If pH is not available, pleural fluid glucose should be measured. It correlates closely to pleural fluid pH. Low pleural fluid glucose concentrations (< 3.4 mmol/L) are indicative of complicated parapneumonic effusion, but are also found in malignancy, tuberculosis and rheumatoid disease. Due to low specificity and poorer diagnostic performances in comparison to pleural fluid pH, the measurement of glucose in pleural fluid should be interpreted in correlation with clinical symptoms (*14*, *18*, *23*).

###### Cholesterol and triglycerides

The combined measurement of pleural fluid cholesterol and pleural fluid triglycerides should be used as part of chylous pleural effusions’ evaluation (*i.e.* to demonstrate lymph presence in pleural fluid sample) (Class 1) (*5*, *18*, *23*, *58*-*60*).

Chylous effusion (or chylothorax) results from chyle or lymph accumulation in the pleural space due to leakage from the thoracic duct or other lymphatic vessels (*i.e.* their disruption or obstruction). The most common causes are malignancy (*e.g.* Hodgkin’s lymphoma) and trauma. Despite the similar appearance, chylous effusions should be differentiated from pseudochylous effusions, due to their different aetiology and treatment approaches. Since chyle is composed of chylomicrons, high triglyceride concentrations are expected. Conversely, pseudochylous (or chyliform) effusions are cholesterol rich, and are caused by chronic pleural inflammation (*e.g.* tuberculous or chronic rheumatoid pleural effusions). Usually, pleural fluid triglycerides and cholesterol are measured simultaneously to exclude pseudochylotorax presence (*5*, *14*, *23*, *24*, *27*, *57*).

Pleural fluid triglycerides ≥ 1.2 mmol/L and pleural fluid cholesterol < 5.2 mmol/L are associated with chylous effusions. Pseudochylous effusions have triglycerides < 1.2 mmol/L, with concomitant cholesterol > 5.2 mmol/L (*18*, *22*, *27*, *56*, *58*).

###### Creatinine

Creatinine should be measured in pleural fluid samples only in cases of suspected urinothorax (*i.e.* to demonstrate the presence of urine in pleural fluid sample). A pleural fluid to serum creatinine ratio > 1 is considered a hallmark of urinothorax, but should always be interpreted in relation to other clinical findings (Class 1) (*5*, *14*, *59*).

Urinothorax (*i.e.* accumulation of urine in the pleural space) is a rare cause of pleural effusion and may be caused by obstruction due to malignancy, fibrosis or calculus, blunt or surgical trauma. Creatinine is considered a sensitive and specific indicator of urine leakage presence. The urinothorax fluid sample is usually transudative (based on low protein concentrations measured in such pleural fluid samples), but can erroneously be classified as exudative if high LD activities are measured in the same sample. This must be taken into account when interpreting Light’s criteria in correlation with high clinical suspicion for urinothorax. However, urinothorax is characterized with markedly elevated creatinine concentrations in the pleural fluid sample (177 - 884 μmol/L) in comparison to creatinine concentrations found in simultaneously collected serum samples. Furthermore, pleural fluid to serum creatinine ratio is considered the biochemical criterion for urinothorax diagnosis: in case of urinothorax its value is > 1 (*5*, *58*-*61*).

###### Tumour markers

The routine determination of single tumour markers or their combinations in diagnosis of malignant pleural effusions is not recommended. Tumour markers should be measured in pleural fluid in cases of inconclusive cytology results (Class 1) (*38*, *62*).

Cytological examination of pleural fluid samples yields high diagnostic specificity and low diagnostic sensitivity (*i.e.* 50-60%) in differentiating malignant causes of pleural fluid accumulation. Various tumour markers have been investigated for the purpose of improving differentiation of malignant from non-malignant effusions including carcinoembryonic antigen (CEA), cancer antigen (CA) 19-9, CA 125, cytokeratin 19 fragment (CYFRA 21-1), CA 15-3, neuron specific enolase (NSE) *etc.* However, their usefulness is limited due to low sensitivity (*5*, *14*, *18*). If malignant pleural effusion is suspected with negative cytological findings, or in case of unknown primary source of malignancy, pleural fluid tumour markers may be helpful as a complementary diagnostic tool to pleural biopsy (*14*, *62*, *63*).

#### 3.1.3 Postanalytical phase

Test reports should include the test result and the type of fluid analysed (*5*, *10*). Laboratories should provide clinical decision limits and interpretive information with each pleural fluid test result to guide clinical interpretation and decision-making (Table 3 and Appendix 2) (Class 1) (*2*, *5*, *18*, *23*).

**Table 3:** Example of recommended reporting format for test results

**Test/Analyte**	**Result**	**Unit**	**Decision limits/Interpretation**
(PF) Appearance		/	Transudates are clear, light yellow, odourless, nonviscous. Exudates are cloudy, turbid, milky, bloody, with clotting tendency
PF/serum protein ratio		/	Exudative effusions meet at least one the following criteria: 1. pleural fluid/serum protein ratio > 0.5; 2. pleural fluid/serum LD ratio > 0.6 3. absolute pleural fluid LD activity > 2/3 of serum URL.
PF/serum LD ratio		/	
(PF) LD		U/L	
Albumin gradient		g/L	Transudates have an albumin gradient > 12 g/L, while exudates have an albumin gradient ≤ 12 g/L.
PF cholesterol		mmol/L	Exudates have a cholesterol >1.2 mmol/L.
PF/serum cholesterol ratio		/	Exudates have a PF/serum cholesterol ratio > 0.3.
**Comment:** (*e.g.* “Traumatic tap. The possible interference on test results cannot be ruled out.”) **Interpretive comment:** (*e.g.* “The analyses performed suggest the presence of a transudative/exudative effusion.” or “The possible interference of matrix differences cannot be excluded; results should be interpreted in relation to the clinical context.”)			
This table represents an exemplary reporting format for tests performed in pleural fluid samples. The template might be customized (and expanded) according to the needs of each individual laboratory, depending on the most prevalent EBF types and tests. PF – pleural fluid. LD – lactate dehydrogenase. URL – upper reference limit.			

If assays used in pleural fluid analysis have not been validated, this should be clearly stated (commented) on the test report and the ordering clinician should be contacted to explain these limitations (see example in Table 3) (Class 1) (*2*, *5*, *17*).

Laboratories should validate postanalytical pleural fluid stability in order to determine the storage period in which additional testing or retesting is feasible (Class 1) (*2*, *64*).

Laboratories are strongly encouraged to communicate and comment the results obtained by pleural fluid analysis with responsible clinical personnel in order to aid diagnosis, patient management or advise on further analysis. Standardized interpretive comment should be included on the pleural fluid analysis test reports (Table 3) (Class 1) (*21*, *65*, *66*).

Since pleural effusions do not accumulate in healthy people and procedure invasiveness prevents collection from a reference population, the lack of appropriate reference ranges to use for comparison as interpretive guidance is considered the most important postanalytical issue in pleural fluid analysis. Pleural fluid analysis results should be reported compared to results from a simultaneously obtained serum sample for the same assay, in order to facilitate results interpretation (*2*, *5*, *17*, *22*).

Clinical decision limits for clinically useful analytes in pleural fluid analysis are provided throughout this document and should be implemented in results interpretation for specific EBF types (Appendix 2).

According to available literature data, total protein and albumin in pleural fluid samples are stable at room temperature (at 21–25°C), at 4°C and at - 20°C for 7 days. Pleural fluid cholesterol is stable for 4 days at room temperature, and for 14 days at 4°C and - 20°C. Pleural fluid triglycerides are stable for 4 hours at all three temperature conditions indicated. Glucose in pleural fluid samples is stable for 2 hours at room temperature, and 7 days at 4°C and - 20°C. The stability of pleural fluid LD is limited to 4 hours at room temperature and 1 day at 4°C, whereas due to LD instability at - 20°C, pleural fluid samples should not be frozen (*2*, *28*, *64*). If the determination of WBC and differential cell count is needed, pleural fluid samples taken with EDTA might be stored for up to 2 days at 4°C (*67*). Ideally, laboratories should validate the stability of analytes in pleural fluid samples in their own routine setting (*2*, *5*).

Interpretive comments might address the preanalytical and analytical phase of pleural fluid analysis. Only the inclusion of interpretive comments which add clinical value should be considered (*e.g.* potential implications of results, further analyses that might address differential diagnosis). The comments should be standardized (predefined) by the laboratory, written in clear and unambiguous language (Table 3). If the LIS is used to generate standardized comments for pleural fluid analyses, relevant literature references should be listed in the test report. If comments are added to the test report as free text, the person responsible for commenting should be clearly stated on the test report. Interpretive comments on test report should not exclude the practice of directly communicating and interpreting results with responsible clinical personnel (*65*, *66*).

### 3.2 Pericardial fluid

**Figure 2:**
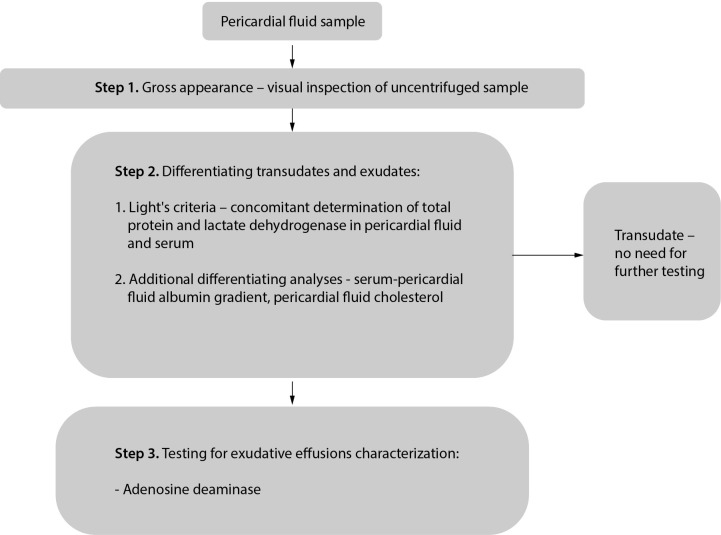
Algorithm for pericardial fluid testing.

Small volumes of pericardial fluid (15-50 mL) fill the pericardial cavity and allow the heart to easily move during contraction and relaxation. The most frequent cause of pericardial effusions is acute pericarditis of bacterial, tuberculous or fungal origin. Furthermore, pericardial effusions can be associated with myocardial infarction, malignancy, uraemia or mediastinal injury (*5*, *6*, *18*, *22*). Contrary to well-established analysis pleural effusions, the utility of tests in pericardial effusions’ evaluation has not been extensively investigated primarily due to the invasive nature of the collection process. Pericardiocentesis, the removal of pericardial fluid for diagnostic or therapeutic purposes, is indicated in cases of large/moderate undiagnosed pericardial effusions; when purulent, tuberculous or malignant pericarditis is suspected; or hemodynamic instability is present leading to cardiac tamponade. Laboratory evaluation of pericardial fluid initiates with the differentiation of transudative from exudative effusions. In general, this approach simplifies the diagnostic process, and the identification of transudative effusions indicates an underlying systemic disease. This excludes the need for further laboratory diagnostic workup (Figure 2) (*18*, *23*, *68*).

#### 3.2.1 Preanalytical phase

The preanalytical recommendations pertaining to pleural fluid test request form and test ordering, patient and sample identification, sample collection and handling, and sample quality assessment are transferable to pericardial fluid testing and should be applied (Class 1).

#### 3.2.2 Analytical phase

##### 3.2.2.1 Pericardial fluid appearance

Pericardial fluid appearance should be determined upon sample acceptance and before centrifugation. Pericardial fluid appearance should not be used as a definitive test to differentiate transudates from exudates (Class 1) (*6*, *22*, *23*).

Normal pericardial fluid is a clear and light yellow fluid, while turbid (serosanguinous) fluids are produced in infections or malignancies. Bloody pericardial fluid may be caused by cardiac rupture or puncture of a ventricle during pericardiocentesis, or traumatic pericardiocentesis. Milky pericardial fluid appearance suggests the presence of chylopericardium (*6*, *22*, *23*).

##### 3.2.2.2 Differentiating transudates and exudates

Pleural fluid cut-off points for Light’s criteria, pleural fluid albumin gradient and pleural fluid cholesterol should be used in discriminating pericardial fluid exudates from transudates. The limited evidence available describing the use of Light’s criteria (using the same cut-offs as for pleural fluid), demonstrated good diagnostic performances (Figure 2, Appendix 2). However, results should always be interpreted in correlation with clinical symptoms (Class 2) (*18*, *69*, *70*).

Similar to pleural effusions, the highest misclassification rates of transudates as exudates were found in patients receiving diuretics. Albumin gradient ≤ 12 g/L was used to identify pericardial fluid exudates, yielding a sensitivity and specificity of 90% and 89%, respectively. An exudative pericardial fluid cholesterol cut-off point of ≥ 1.6 mmol/L yielded a sensitivity and specificity of 71% and 83%, respectively (*71*). Another investigation found that although the composition of normal pericardial fluid was similar to that of serum/plasma, pericardial fluid LD activities were 2.4 times higher compared to serum activities and protein concentrations were 0.6 of serum concentrations. Thus, the utility of measuring pericardial fluid total protein and LD is limited because transudative pericardial fluid samples meet Light’s exudative criteria (for pleural fluid classification) (*69*). Furthermore, total cell count and differentials, LD, protein and glucose, as sole analyses or as part of calculated ratios, yielded poor discriminative properties in identifying the cause of pericardial effusion (*72*).

##### 3.2.2.3 Analysis of exudative pericardial effusions

Pericardial fluid ADA activities should be determined in identifying tuberculous pericarditis. The proposed ADA cut-off for tuberculous pericarditis is ≥ 40 U/L (Appendix 2) (Class 2) (*73*).

Analysis of exudative pericardial effusions is most commonly focused towards the differentiation of malignant from non-malignant effusions (by cytological examination) and/or the confirmation of a specific diagnosis that caused the effusion (*e.g.* infection) (*6*). Current scientific evidence pertaining to laboratory evaluation of pericardial exudates is limited. Pericardial effusion evaluation might include cell count, glucose, total protein and LD (*70*). Both total and differential cell count are of limited diagnostic value in the assessment of pericardial effusions. Total leukocyte count > 10 x10^9^/L suggests bacterial, tuberculous or malignant pericarditis (*6*, *23*, *70*).

The measurement of pericardial fluid pH is of no clinical value (*74*). Chylous and pseudochylous pericardial effusions may be separated by triglyceride and cholesterol measurement in pericardial fluid samples. The decision limits for pleural fluid may be applied for this purpose (*6*, *22*, *70*).

#### 3.2.3 Postanalytical phase

The postanalytical recommendations pertaining to pleural fluid analysis are transferable to pericardial fluid testing and should be applied (Class 1).

### 3.3 Peritoneal fluid (ascites)

The peritoneal space is a mesothelial lined space which physiologically contains up to 50 mL of peritoneal fluid formed by the ultrafiltration of plasma (*23*). Peritoneal effusion (ascites) refers to the pathological accumulation of fluid in this cavity due to increased fluid formation or decreased fluid removal. The most frequent causes of ascites are hepatic cirrhosis, malignancy, heart failure, tuberculosis, nephrotic syndrome, bacterial peritonitis and pancreatitis (*18*, *23*, *75*-*77*).

Radiological, ultrasound and computed tomography (CT) studies are procedures that allow detection of small volumes of peritoneal fluid and are helpful in assessing the possible cause of ascites. However, diagnostic paracentesis is considered essential in all patients with ascites prior to therapeutic interventions to exclude spontaneous bacterial peritonitis (SBP) and causes of ascites other than cirrhosis. Furthermore, diagnostic paracentesis is indicated in patients with new-onset ascites, in patients requiring hospitalisation due to ascites and in those with ascites accompanied with unexplained clinical worsening (*78*-*81*). Analysis of peritoneal fluid is considered a cost-effective and rapid method in establishing ascites aetiology (*23*, *27*, *82*).

#### 3.3.1 Preanalytical phase

Preanalytical recommendations pertaining to pleural fluid test request form and test ordering, patient and sample identification, sample collection and handling, and sample quality assessment are transferable to ascites testing and should be applied (Class 1).

#### 3.3.2 Analytical phase

##### 3.3.2.1 Ascites appearance

Ascites appearance should be determined upon sample acceptance and before centrifugation. Ascites appearance should be used as an aid in elucidating its aetiology, not as the sole criterion for differential diagnosis of fluid accumulation (Class 1) (*21*, *23*, *77*).

Normally, ascites is clear and pale-yellow. If bloody appearance is caused by traumatic tap, the fluid tends to clot when left standing after collection and eventually clears up. The persistence of milky appearance after centrifugation indicates the presence of lymph (*i.e.* chylous or pseudochylous effusion abundant in chylomicrons with high triglycerides concentrations); purulent ascites is associated with intra-abdominal infection. The possible interpretation of peritoneal fluid appearance is presented in Table 4 (*23*, *27*, *75*).

**Table 4:** Possible interpretation of peritoneal fluid appearance

**Appearance**	**Possible clinical significance**	**References**
Clear, pale yellow	Cirrhosis, no need for further laboratory testing	21,23,27,75,77
Deep yellow, detergent-like	Possible bilirubin presence, jaundice	
Milky	Chylous or pseudochylous ascites present in cirrhosis, infections, malignancy, congenital defects	
Bloody	Malignancy, tuberculous peritonitis, abdominal trauma, pancreatitis	
Turbid, purulent	Bacterial peritonitis, pancreatitis or malignancy	
Dark brown (tea-coloured)	Pancreatic ascites	
Black	Haemorrhagic pancreatitis, malignant melanoma	
Dark, molasses coloured appearance	Gut perforation	
Green, brown	Bile presence, gallbladder perforation, intestine perforation, duodenal ulcer, cholecystitis, acute pancreatitis	

##### 3.3.2.2 Differentiating peritoneal effusions

**Figure 3:**
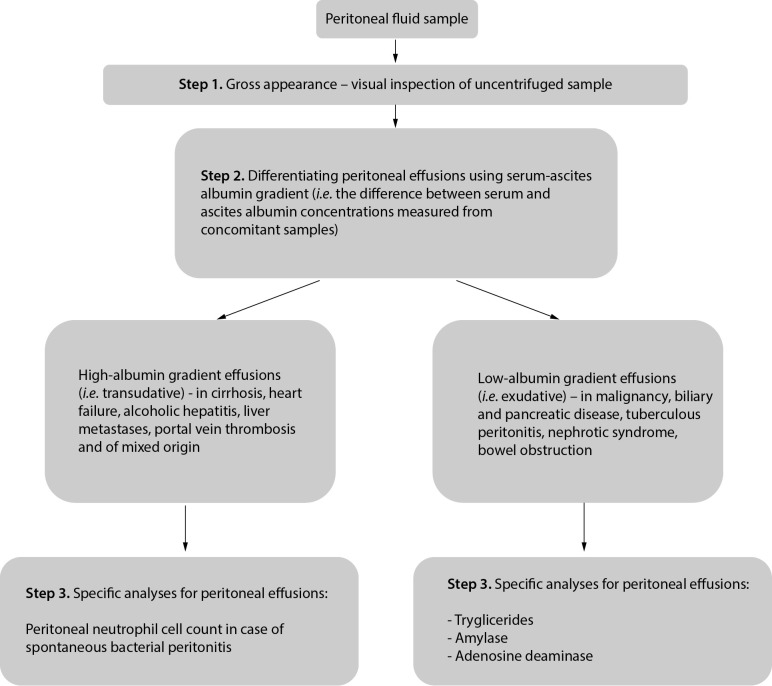
Algorithm for peritoneal fluid testing

The serum-ascites albumin gradient (SAAG), *i.e.* the difference between serum and ascites albumin concentrations, should be used for differentiation of peritoneal effusions caused by portal hypertension and those caused by other pathophysiological mechanisms. Peritoneal effusions with SAAG ≥ 11 g/L should be classified as high-albumin gradient effusions and considered transudative. Alternatively, peritoneal effusions with SAAG < 11 g/L should be classified as low-albumin gradient effusions, *i.e.* exudative (Figure 3) (Class 1) (*21*, *23*, *78*-*80*, *82*-*84*).

The traditional transudate/exudate concept for peritoneal effusion differentiation, based on the assumption that high ascites total protein concentrations are helpful in identifying exudates, should be abandoned. In fact, many malignant and infectious peritoneal effusion samples were misclassified as transudates; while effusions from patients with cirrhosis and heart failure were misclassified as exudates, when the traditional total protein cut-offs ranging from 25-30 g/L were applied. Moreover, peritoneal effusions collected from healthy women showed total protein concentrations in the range of 40 g/L, placing them in the exudative range. Finally, this traditional concept does not take account for mixed ascites (*i.e.* ascites due to combination of portal hypertension and another disorder) (*21*, *23*, *75*, *77*, *84*).

Poor diagnostic performances of total protein concentrations led to the investigation of more useful diagnostic parameters (and their combinations) for peritoneal fluid differentiation (*23*, *84*, *85*). For example, Boyer’s criteria which include the measurement of total protein and LD in ascites and serum, ascites/serum bilirubin ratio, cholesterol in ascites, ascites/serum cholesterol ratio and a model combining concentrations of total protein, LD, tumour necrosis factor α (TNF-α), complement C4 and haptoglobin measured in ascites, have been proposed for identification of exudative peritoneal effusions. However, the lack of reproducible evidence has precluded their utilization in practice (*23*, *75*).

The SAAG is independent of peritoneal membrane permeability, reflects the presence/absence of portal hypertension and is considered the physiological alternative to the traditional transudate/exudate concept. If portal hypertension is the cause of peritoneal fluid accumulation, the osmotic gradient between serum and ascites will be increased in order to compensate for high hydrostatic pressure. Using a SAAG cut-off of 11 g/L, peritoneal effusions can be differentiated to those related to portal hypertension (*i.e.* high albumin-gradient effusions in case of cirrhosis, heart failure, alcoholic hepatitis, liver metastases, portal vein thrombosis and ascites of mixed origin), and those related to normal portal pressure (*i.e.* low albumin-gradient effusions in cases of malignancy, biliary and pancreatic ascites, tuberculous peritonitis, nephrotic syndrome, bowel obstruction) (*23*, *75*, *79*, *83*, *84*, *86*). Compared to total protein concentrations, SAAG yields a diagnostic accuracy of 97% (sensitivity of 95% and specificity of 95%) in the identification of exudative ascites (*77*, *78*, *82*). This difference in diagnostic performances might be explained by the fact that SAAG correlates directly to portal hypertension, while total proteins are inversely correla-ted to portal pressure but directly to serum total proteins (*23*, *77*, *82*, *84*).

Despite the superiority of SAAG, its values should be interpreted with caution considering several albumin methodological issues. Albumin concentrations (in serum and ascites samples) might be determined by using spectrophotometric (bromcresol green) methods. Alternatively, nephelome-tric methods might be used. Measuring albumin concentrations in the lower concentration range is analytically challenging. Patients with cirrhosis might exhibit very low serum albumin concentrations (*i.e.* < 15 g/L), which might result in incorrectly low SAAG calculation. Spectrophotometric methods for albumin determination overestimate albumin concentrations in the lower concentration range, compared to immunochemical methods (*23*, *27*, *75*). Bromcresol green methods for albumin determination are prone to transferrin and lipoprotein interference in the low concentration range, which particularly pertains to peritoneal fluid analysis (*e.g.* in chylous ascites, due to lipoprotein interference, albumin concentrations might be falsely overestimated). Furthermore, globulin concentrations contribute to oncotic pressure but are inversely correlated to albumin; thus, the presence of hyperglobulinemia (> 50 g/L) might cause falsely low SAAG (*23*, *87*). Albumin is sensitive to various preanalytical factors, such as posture, prolonged tourniquet stasis, use of diuretics (*76*, *88*). Since the magnitude of the effect of time interval elapsed between serum sampling and peritoneal fluid collection on the SAAG calculation is still poorly understood, the two sample types should be collected simultaneously (*27*, *75*).

##### 3.3.2.3 Specific analyses for peritoneal effusions

###### Peritoneal fluid neutrophil count

Peritoneal fluid neutrophil count ≥ 250 x10^6^/L, in the absence of perforated or inflamed intra-abdominal organs, is the key criterion to support the diagnosis of SBP. It should be determined in all hospitalized patients with cirrhosis accompanied with ascites for SBP exclusion (*21*, *23*, *48*, *78*-*81*). The neutrophil cell count in peritoneal fluid samples should be determined using automated haematology analysers, or alternatively manual microscopy (*i.e.* using a haemocytometer) (Class 1).

Spontaneous bacterial peritonitis is defined as an infection of pre-existing peritoneal (ascitic) fluid in the absence of any other intra-abdominal source of infection. It is a frequent complication in cirrhotic patients with ascites and is caused by translocation of bacteria from the intestine into the peritoneal cavity. Its prevalence in cirrhotic hospitalised patients with ascites is estimated between 10-30%. Although high total WBC in ascitic fluid provides quick information on the presence of infection and is still determined in the diagnosis of SBP irrespective of differential count (diagnostic cut-off > 500 x10^6^/L), ascitic neutrophil counts should be used when diagnosing SBP. Neutrophil count cut-off values of 250 x10^6^/L and 500 x10^6^/L for SBP diagnosis have similar diagnostic accuracies; however the former cut-off displays better sensitivity, while the later better specificity. If the peritoneal sample is grossly bloody (with a fluid red blood cell count > 10 x10^9^/L), one neutrophil should be subtracted from the neutrophil absolute count every 250 red blood cells to adjust for the presence of blood (*e.g.* in a sample with neutrophil count of 250 x10^6^/L and red blood cell count 20 x10^9^/L; the adjusted neutrophil count is 170 x10^6^/L) (*21*, *48*, *78*-*82*, *89*).

###### Peritoneal fluid neutrophil count using urine test strips

Since SBP can be a life-threatening condition, early detection and quick therapeutic decisions are of paramount importance in reducing mortality rates in hospitalized patients. Accordingly, urine strips (*i.e.* the leukocyte esterase test) have been proposed as rapid and available tool for the early detection of high neutrophil counts in peritoneal fluid. However, urine strips displayed low sensitivity and high false negative results, especially in cases of SBP and low neutrophil count. Therefore, they are not recommended for the rapid diagnosis of SBP. The advances in automated technologies for cell counting and differentials in EBF have prevailed over qualitative methods like urine strips (*90*-*95*).

###### Other peritoneal fluid analyses in diagnosing SBP

Total proteins in peritoneal effusions might be determined to assist the estimation of the risk for SBP development. Total protein concentrations < 10 g/L have been associated with greater risk of developing SBP. Ascitic LD activities are high in SBP and secondary bacterial peritonitis (*75*, *81*, *96*).

Calprotectin is a protein originating from neutrophils. Its higher concentrations have been found in plasma and stool samples of patients with infectious and inflammatory conditions. Ascitic calprotectin has been suggested as a novel sensitive and specific indicator for detection of SBP in cirrhotic patients with ascites. It might be quantified using a commercially available point of care test and is a reliable alternative method for predicting peritoneal fluid neutrophil counts > 250 x10^6^/L. Due to limited data on its diagnostic utility, it has not yet been widely accepted in routine practice (*97*-*99*).

###### Triglycerides

Triglycerides measurements in peritoneal effusions should be performed when the presence of chylous ascites is suspected (*i.e.* to demonstrate the presence of lymph in peritoneal samples). Ascitic/serum triglycerides ratio > 1 or ascites triglycerides concentrations > 1.2 mmol/L are used for the identification of chylous ascites (Class 1) (*100*, *101*).

Chylous ascites formation is related to obstruction and/or injury of the intestinal lymphatic system and the accumulation of lymphatic fluid in the peritoneum. Pseudochylous ascites occurs due to cell degradation in bacterial peritonitis or malignancy. Chylous ascites is differentiated from pseudochylous effusions by the finding of triglycerides concentrations higher than those measured in serum (ascitic/serum triglycerides ratio > 1 or ascites triglycerides > 1.2 mmol/L). Since chylous ascites is usually rich in tryglicerides, the additional measurement of cholesterol in ascites is not necessary. Triglycerides concentrations depend on patient nutritional status, it is imperative that the serum sample is collected simultaneously (*23*, *75*, *102*, *103*).

###### Amylase

Amylase activity in peritoneal effusions should be measured solely when confirmation or exclusion of pancreatic ascites, gut perforation, ruptured pseudocysts and mesenteric thrombosis is needed. The highest activities (*i.e.* three times the normal serum value or ≥ 2000 U/L) are usually associated with pancreatic damage (Class 1) (*21*, *23*, *75*, *78*).

###### Adenosine deaminase

Peritoneal effusion ADA activities are a sensitive and specific indicator of tuberculous ascites and should be measured for its confirmation, especially in high prevalence areas. An optimal cut-off of ≥ 39 U/L has high diagnostic accuracy for the diagnosis of tuberculous peritonitis (with sensitivity of 100% and specificity of 97%) (Class 2) (*104*).

###### Other specific analyses

Peritoneal fluid urea, creatinine, total bilirubin, glucose and cholesterol might be measured in specific clinical situations. However, limited evidence available on their added diagnostic utility limits their inclusion in the recommended analyses (*75*, *80*). Peritoneal fluid cholesterol concentrations might be useful in the differentiation of malignant ascites from other ascites aetiologies (*e.g.* due to cirrhosis). Higher cholesterol concentrations are inherent to malignant ascites due to increased permeability, cholesterol synthesis and cholesterol release form malignant cells. An ascites cholesterol cut-off of > 1.2 mmol/L yielded a diagnostic sensitivity of 93% and diagnostic specificity of 96% for the differentiation of malignant ascites (*18*, *75*, *105*, *106*).

Glucose concentrations measured in peritoneal effusions mirror those found in serum. Consequently, the measurement of ascitic glucose concentrations has little clinical value, except when the presence of infection or malignancy is suspected (*e.g.* tuberculous peritonitis, carcinomatosis or SPB). Low glucose concentrations (*e.g.* < 2.8 mmol/L) found in peritoneal effusions might indicate its increased consumption in the presence of leukocytes and bacteria (*21*, *23*, *75*, *82*).

Urine leakage from the urinary tract into the peritoneal cavity presents usually as a transudate with high creatinine and urea concentrations, and low glucose and pH. The determination of urea and creatinine in peritoneal effusions is useful in the differentiation of urine from peritoneal fluid (*i.e.* to confirm urine presence in the ascites sample). Ascitic creatinine and urea concentrations higher than in a concomitant serum sample (with fluid to serum creatinine ratio > 1) suggest urinary bladder rupture (*5*, *21*, *23*).

Total bilirubin should only be measured when the ascitic sample is brown-coloured. Peritoneal fluid bilirubin concentrations higher than those found in a concomitant serum sample are indicative of biliary leak (*e.g.* in intrahepatic or gallbladder fistula, gut perforation) (*5*, *75*, *96*).

Secondary bacterial peritonitis, which develops after perforation of peptic ulcer or in case of perinephric abscess, is an ascitic infection characterized with positive bacterial culture with ascitic fluid neutrophils < 250 x10^6^/L. It should be differentiated from SBP to promptly initiate appropriate therapy to reduce mortality. Analysis of ascitic fluid might help in the diagnosis of secondary bacterial peritonitis using the following criteria (at least two criteria should be met): total protein > 10 g/L, glucose < 2.8 mmol/L and LD > the URL for serum (*18*, *96*, *103*).

Ascitic fluid pH and lactate are insensitive and nonspecific tests for detection of ascitic fluid infection and should not be measured (*96*, *103*).

#### 3.3.3 Postanalytical phase

The postanalytical recommendations pertaining to pleural fluid analysis are transferable to peritoneal fluid testing and should be applied (Class 1).

## Appendix 1

### Cell counting in serous fluids analysis

Cells found in serous fluids include WBC, erythrocytes, nucleated erythrocytes, lining cells (*i.e.* mesothelial) and malignant cells. The determination of total cell counts and differentials in EBF samples is clinically important in infectious, inflammatory, haemorrhagic and malignant disorders affecting body cavities. Traditionally, counting and differentiating cells in serous fluids has been performed manually in a haemocytometer. Nowadays, this technique is rapidly being replaced with automated cell enumeration and differentiation. This section addresses available cell counting methodologies with their main advantages and limitations (*13*, *15*, *48*).

#### Manual cell counting

Each laboratory should have a documented operating protocol for manual cell counting depending on the haemocytometer (counting chamber) used. Two types of counting chambers are most widely used: the Fuchs-Rosenthal and the Neubauer Improved chamber. Both use the same counting principle but differ in dimensions (depth and counting area) which should be taken into account for accurate calculations of the final cell count (Table A). The sample should be well mixed before analysis (tube inversion 10-15 times), especially if turbid. The haemocytometer should be loaded on both sides with usually one drop of the cell suspension, being careful not to overfill, but to cover the entire surface of the counting grid. Cells should be allowed to settle for 5 minutes after loading and then counted as soon as possible. If the sample draws back from the sides of the haemocytometer, the sample has dried out and the haemocytometer should be loaded again to avoid erroneous results (*13*, *15*). The counting chamber is placed under the microscope, low magnification (10x) is applied to adjust the focus and inspect the counting area. Accurate counting is obtained if cells are evenly distributed and do not overlap. High magnification (40x) is then applied and cells are counted. The extent of the cell counting area depends on the number of cells present in the preparation and the characteristics of the chamber used (Table A). Erythrocytes and nucleated cells are usually counted in the same chamber in undiluted samples. However, if samples are bloody or turbid, they should be diluted (from 1:10 to 1:200 or higher) using isotonic saline (for erythrocytes and WBC dilutions) or diluted acetic acid and/or hypotonic saline (to lyse erythrocytes for WBC dilutions). If samples are diluted, a minimum of 200 cells should be counted. Each laboratory should include a defined dilution protocol in the laboratory’s manual cell operating protocol (*13*, *15*).

**Table A:** Main characteristics of the Neubauer Improved and Fuchs-Rosenthal haemocytometers

	**Neubauer Improved haemocytometer**	**Fuchs-Rosenthal haemocytometer**
**Counting grid**	The whole counting grid is 3 mm x 3 mm in size (total area of 9 mm^2^). It is divided in 9 squares, each 1 mm wide, and each square is further sub-divided in 16 squares (0.25 x 0.25 mm in size). The central square is divided in 25 squares (sized 0.20 x 0.20 mm), which are further divided in 16 smaller squares (sized 0.05 x 0.05 mm). The depth is 0.1 mm.	The whole counting grid is 4 mm x 4 mm in size (total area of 16 mm^2^). It is divided in 16 squares, each 1mm wide, and each square is further sub-divided into 16 squares (0.25 mm wide). The depth is 0.2 mm.
**Counting area**	If less than 200 cells are estimated in all 9 squares, count all nine squares (area = 9 mm^2^). If more than 200 cells are estimated in all 9 squares, the four corner squares should be counted (area = 4 mm^2^). If more than 200 cells are estimated in one square, cells should be counted in the five center squares (area = 0.2 mm^2^).	If less than 200 cells are estimated in all 16 squares, count all 16 squares (area = 16 mm^2^). If more than 200 cells are estimated in all 16 squares, the four corner squares should be counted (area = 4 mm^2^). If more than 200 cells are estimated in one square, cells should be counted in one of the center squares (area = 1 mm^2^).
**Calculations**	Cells (x10^6^/L) = (number of cells counted x dilution factor) / (number of mm^2^ counted x chamber depth)	Cells (x10^6^/L) = (number of cells counted x dilution factor) / (number of mm^2^ counted x chamber depth)
Adapted from (*13*, *21*).		

The final cell count represents the average of counts from each side of the chamber. The limits of agreement of two separate counts should be defined by each laboratory (usually not exceeding 20%) (*13*, *15*). Results should be reported as absolute values of nucleated cell and erythrocytes in conventional units (x10^6^/L) (*13*, *15*).

Differential cell counts of serous samples should be performed on stained smears prepared using cytocentrifugation (*21*). Cytospin smears permit cell concentration while minimizing cell deformation. Since serous fluids could contain fibrin and other proteins which may occlude the filter in the cytocentrifuge, aliquots of the sample might be centrifuged and cells re-suspended in saline prior to cytocentrifugation. Cytospin smears are stained according to Pappenheim or Wright, and differential counts are then performed on a minimum of 100 cells. Results are reported as counts (expressed as %) of nucleated cells subtypes (*e.g.* neutrophils, eosinophils, lymphocytes, macrophages, other cells, *etc*.). Other cells (comprising mesothelial lining cells, tumour cells, atypical cells) found during cell differentiation should be described in the comment section of the report (*13*, *21*).

Although manual methods are considered gold standards in serous cell counting and differentiation, they have several limitations. Manual microscopic techniques for cell counting and differentiation are highly subjective and time-consuming, require technical expertise, have high inter- and intra-observer imprecision and poor reproducibility. Furthermore, cytocentrifugation might have an effect on cell recovery (*i.e.* susceptible cells may be lost or be subjected to morphological changes; macrophages and mesothelial cells may form clusters and be wrongly attributed to malignant cells) (*13*, *15*, *48*).

#### Automated cell analysis

Automated cell counting methods are rapidly displacing the manual ones in clinical laboratories primarily due to their speed, consistency and reliability. These methods should be methods of choice for counting and differentiating cells in serous fluids. Depending on the automated analyser, available methodologies for counting and differentiating cells include impedance, flow cytometry and flow cell digital imaging. Automated analysers with a separate body fluid mode are designed to take into account differences in cellularity and matrix between body fluids and whole blood. Such analysers are presently widely available and all results provided by automated cell analysis (including erythrocyte count, total nucleated cell count (TNC), WBC count and WBC differentials) should be reported. Automated serous fluid cell analysis should be performed according to manufacturer’s instructions for use and on samples cleared by the manufacturer in the intended use section (*13*, *15*, *48*, *107*).

Current drawbacks of automated body fluid counting methods can be summarized as lack of satisfying precision in the low WBC range, lack of ability to discover malignant cell or non-cellular interferences (*e.g.* bacteria, lipids or crystals) and limited ability to flag abnormalities. Alternative verification methods (including manual cell counting) should be established by each laboratory for samples with results below the lower limit. Similarly, reflex testing rules should be established for flagged results obtained by automated methods (*107*-*113*).

## Appendix 2

### Summary of criteria for serous fluid analysis

**Table:**

**EBF**	**Criterion**	**Interpretation**
**Pleural fluid**	Light’s criteria: 1. pleural fluid/serum protein ratio > 0.5; 2. pleural fluid/serum LD ratio > 0.6 3. absolute pleural fluid LD activity > 2/3 of serum URL	Exudative effusion if at least one criterion is met.
	serum-pleural fluid albumin gradient ≤ 12 g/L	If clinical symptoms suggest transudative pleural effusion, but Light’s exudative criteria are met (usually by a small margin), the albumin gradient (calculated as the serum albumin concentrations minus pleural fluid albumin concentration) should be used as a tool to confirm true transudative pleural effusions. An albumin gradient > 12 g/L is indicative of transudates, while exudates have an albumin gradient ≤ 12 g/L.
	cholesterol > 1.2 mmol/L	A pleural fluid cholesterol cut-off point of > 1.2 mmol/L is accepted for the identification of exudates.
	pleural fluid/serum cholesterol ratio > 0.3	Exudative effusion.
	LD > 0.45 serum URL and cholesterol > 1.2 mmol/L	The paired combination of pleural fluid LD and pleural fluid cholesterol yielded a 98% specificity and 72% sensitivity in identifying exudates. An exudative effusion is identified if both criteria are met.
	protein > 29 g/L, LD > 0.45 serum URL and cholesterol > 1.2 mmol/L	This triplet of pleural fluid tests yielded a sensitivity of 98% and specificity of 70% in discriminating exudates from transudates. An exudative effusion is identified if all criteria are met.
	total WBC count > 1000 x10^6^/L with neutrophil predominance (≥ 50% of total WBC)	Acute pleural inflammation, bacterial pneumonia, pancreatitis, early tuberculosis.
	total WBC count > 1000 x10^6^/L with lymphocyte predominance (≥ 50% of total WBC)	Tuberculosis, viral infection, malignancy, chylothorax, rheumatoid pleuritic.
	total WBC count > 1000 x10^6^/L with eosinophilia (> 10% of total WBC)	Pneumothorax, malignancy, haemothorax, pulmonary infarction, congestive heart failure.
	pleural fluid to serum amylase ratio > 1	Pancreatitis, malignancy, oesophageal rupture, pancreatic pseudocyst, liver cirrhosis, cardiac failure, parapneumonic effusion, ruptured ectopic pregnancy, trauma.
	ADA ≥ 40 U/L	Differentiation of tuberculous and malignant pleuritis.
	pH < 7.20 (and pleural fluid LD > 3 serum URL)	Complicated parapneumonic effusion.
	triglycerides ≥ 1.2 mmol/L and cholesterol < 5.2 mmol/L	Chylous effusions.
	pleural fluid to serum creatinine ratio > 1	Urinothorax.
**Pericardial fluid**	Light’s criteria: 1. pericardial fluid/serum protein ratio > 0.5; 2. pericardial fluid/serum LD ratio > 0.6 3. absolute pericardial fluid LD activity > 2/3 of serum URL	Exudative effusion if at least one criterion is met.*****
	serum-pericardial fluid albumin gradient ≤ 12 g/L	Exudative effusion.*****
	cholesterol > 1.2 mmol/L	Exudative effusion.*****
	ADA ≥ 40 U/L	Tuberculous pericarditis.
**Peritoneal fluid**	SAAG < 11 g/L	Low-albumin gradient effusions (*i.e.* exudative).
	neutrophil count ≥ 250 x10^6^/L	Spontaneous bacterial peritonitis.
	ascitic to serum triglycerides ratio > 1	Chylous ascites.
	peritoneal fluid to serum amylase ratio > 1	Pancreatic ascites, gut perforation, ruptured pseudocysts and mesenteric thrombosis.
	ADA ≥ 39 U/L	Tuberculous peritonitis.
EBF – extravascular body fluid. LD – lactate dehydrogenase. URL – upper reference limit. WBC – white blood cells. ADA - adenosine deaminase. SAAG – serum-ascites albumin gradient. *Due to limited evidence, results should always be correlated with clinical symptoms.		
